# Poly[[μ_2_-1,3-bis­(imidazol-1-ylmeth­yl)benzene][μ_2_-2,2′-dihy­droxy-1,1′-methyl­enebis(naphthalene-3-carboxyl­ato)]zinc]

**DOI:** 10.1107/S1600536811054584

**Published:** 2012-01-07

**Authors:** Yanqiang Peng, Xilian Wei, Dacheng Li, Suna Wang

**Affiliations:** a School of Chemistry and Chemical Engineering, Liaocheng University, Shandong 252059, People’s Republic of China

## Abstract

In the title compound, [Zn(C_23_H_14_O_6_)(C_14_H_14_N_4_)]_*n*_, the Zn^II^ ion is four-coordinated in a distorted tetra­hedral geometry. The 1,3-bis­(imidazol-1-ylmeth­yl)benzene and 2,2′-dihy­droxy-1,1′-methyl­enebis(naphthalene-3-carboxy­l­ate) ligands con­nect the Zn^II^ ions alternately in different directions, forming a layered structure parallel to the *ac* plane. Topological analysis reveals that the whole structure is a (4,4) network. The layers are further assembled into a three-dimensional supra­molecular structure *via* C—H⋯O and C—H⋯π inter­actions.

## Related literature

For background to metal-organic frameworks, see: Luo *et al.* (2009 ▶); Wei *et al.* (2010 ▶). For related structures, see: Wang *et al.* (2011 ▶); Fan *et al.* (2005 ▶); Zhou *et al.* (2008 ▶); Li *et al.* (2010 ▶); Feng *et al.* (2009 ▶); Xu *et al.* (2009 ▶); Batten & Robson (1998 ▶).
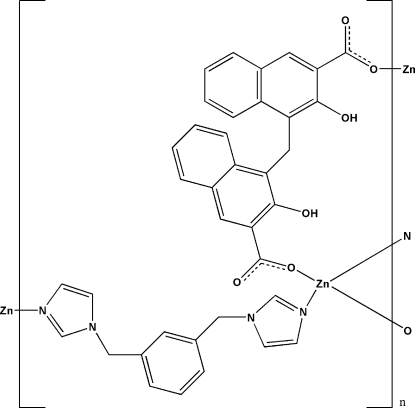


## Experimental

### 

#### Crystal data

[Zn(C_23_H_14_O_6_)(C_14_H_14_N_4_)]
*M* *_r_* = 690.00
Monoclinic, 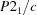

*a* = 10.8382 (9) Å
*b* = 17.3428 (16) Å
*c* = 17.7939 (17) Å
β = 100.781 (1)°
*V* = 3285.6 (5) Å^3^
*Z* = 4
Mo *K*α radiation
μ = 0.80 mm^−1^
*T* = 298 K
0.23 × 0.17 × 0.15 mm

#### Data collection

Bruker SMART-1000 CCD diffractometer
Absorption correction: multi-scan (*SADABS*; Sheldrick, 1996 ▶) *T*_min_ = 0.837, *T*_max_ = 0.889
16511 measured reflections
5800 independent reflections
2460 reflections with *I* > 2σ(*I*)
*R*_int_ = 0.111

#### Refinement

*R*[*F* ^2^ > 2σ(*F* ^2^)] = 0.062
*wR*(*F* ^2^) = 0.127
*S* = 1.00
5800 reflections
433 parameters
H-atom parameters constrained
Δρ_max_ = 0.67 e Å^−3^
Δρ_min_ = −0.68 e Å^−3^

### 

Data collection: *SMART* (Bruker, 2007 ▶); cell refinement: *SAINT* (Bruker, 2007 ▶); data reduction: *SAINT*; program(s) used to solve structure: *SHELXS97* (Sheldrick, 2008 ▶); program(s) used to refine structure: *SHELXL97* (Sheldrick, 2008 ▶); molecular graphics: *SHELXTL* (Sheldrick, 2008 ▶); software used to prepare material for publication: *SHELXTL*.

## Supplementary Material

Crystal structure: contains datablock(s) I, global. DOI: 10.1107/S1600536811054584/vm2140sup1.cif

Structure factors: contains datablock(s) I. DOI: 10.1107/S1600536811054584/vm2140Isup2.hkl

Additional supplementary materials:  crystallographic information; 3D view; checkCIF report

## Figures and Tables

**Table 1:** Hydrogen-bond geometry (Å, °) *Cg*1, *Cg*2 and *Cg*3 are the centroids of the C8–C13, C18–C23 and C4–C9 rings, respectively.

*D*—H⋯*A*	*D*—H	H⋯*A*	*D*⋯*A*	*D*—H⋯*A*
O3—H3⋯O2	0.82	1.81	2.555 (5)	150
O6—H6⋯O5	0.82	1.76	2.502 (5)	150
C30—H30*B*⋯O6^i^	0.97	2.54	3.350 (7)	141
C26—H26⋯*Cg*1^ii^	0.93	2.79	3.676 (8)	161
C29—H29⋯*Cg*2^iii^	0.93	2.76	3.502 (7)	137
C30—H30*A*⋯*Cg*3^ii^	0.97	2.71	3.628 (6)	158

Symmetry codes: (i) 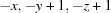; (ii) 
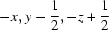
; (iii) 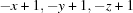.

**Fig. 1.**
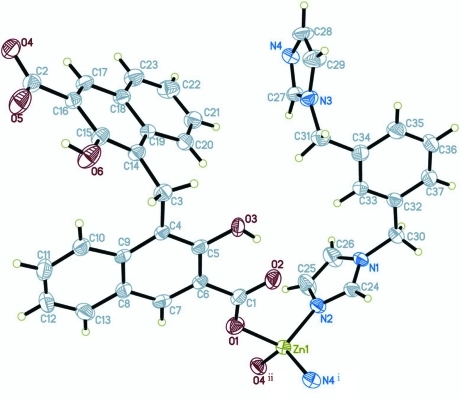
ORTEP drawing of the asymmetric unit of the title compound with 30% probability displacement ellipsoids. Symmetry codes: (i) x - 1, y, z. (ii) x - 1, -y + 3/2, z - 1/2.

**Fig. 2.**
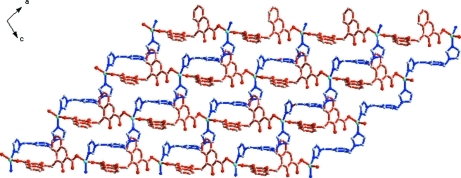
View of the two-dimensional layer of the title compound along the ac plane. PA2- and m-bix ligands are shown in red and blue, respectively.

**Fig. 3.**
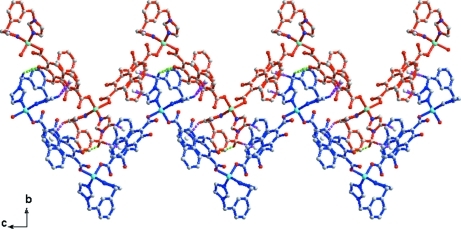
View of the three-dimensional supramolecular network, showing the weak interactions between adjacent layers. C-H···O and C-H···π interactions are represented by green and pink dashed lines, respectively.

**Fig. 4.**
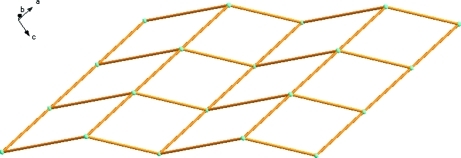
View of the (4, 4) network of the title compound, considering the ZnII ions as four-connected nodes.

## supplementary crystallographic information

### Comment

In the past several years, the design and construction of metal-organic frameworks (MOFs) have received more attention due to their intriguing architectures and potential applications in ion-exchange, heterogeneous catalysis and gas storage (Luo *et al.*, 2009; Wei *et al.*, 2010). Much interest was focused on the coordination chemistry of semirigid polycarboxylate ligands and flexible exo-bidentate N-heterocycle ligands (Wang *et al.*, 2011; Fan *et al.*, 2005; Zhou *et al.*, 2008; Li *et al.*, 2010; Feng *et al.*, 2009; Xu *et al.*, 2009). Herein, we selected pamoic acid (H_2_PA, 4,4^'^-methylenebis(3-hydroxy-2-naphthalenecarboxylic acid)) as building unit. Coexistence of naphthalene rings and the central sp^3^ carbon atom makes this symmetrical aromatic dicarboxylate ligand possess both rigid and flexible character. Solvothermal reactions of this ligand with m-bix (1,3-bis(imidazol-1-ylmethyl)benzene) and Zn^II^ salt led to the title compound, [Zn(PA)(m-bix)]_n_.

The title compound crystallizes in the space group P2_1_/c and exhibits a two-dimensional layered structure. The asymmetric unit is composed of one crystallographically independent Zn^II^ ion, one PA^2-^ anion ligand as well as one m-bix ligand. The Zn^II^ ion is four-coordinated by two carboxylate oxygen atoms from two different PA^2-^ ligands and two nitrogen atoms from two m-bix ligands. The Zn-O and Zn-N bond lengths lie in the normal range of 1.957 (4)-2.009 (5)Å, while the O···Zn···N and O···Zn···O angles are in the range of 98.40 (16)-128.6 (2)°, indicating a much distorted tetrahedral coordination geometry around the metal center. As shown in Fig.1, m-bix ligand in this case adopts a bis(monodentate) bridging coordination mode, linking Zn^II^ ions to form one-dimensional chains along the *a* axis with a dihedral angle between the two terminal imidazole rings of 49.5 (7)°. These chains are connected further by the deprotonated PA^2-^ ligand in *trans* conformation bis(monodentate) bridging coordination mode with the dihedral angle between two naphthyl rings of 97.1 (9)°. As a result, a two-dimensional corrugated layer structure is generated along the *ac* plane (Fig. 2). The metal···metal distances separated by m-bix and PA^2-^ ligands are 15.27 (5) and 10.83 (8)Å, respectively. From a topological viewpoint, the whole structure is a (4, 4) network (Batten *et al.*, 1998) considering the Zn^II^ ions as four-connected nodes (Fig. 4). The carboxyl groups and adjacent hydroxyl groups are linked by intramolecular O-H···O hydrogen bonds. Adjacent layers are further stacked through C-H···O and C-H···π weak interactions, resulting in the present three-dimensional supramolecular structure (Fig. 3 and Table 1).

### Experimental

A mixture of Zn(NO_3_)_2_.6H_2_O(0.1mmol), H_2_PA(0.1mmol), m-bix(0.1mmol), DMF(6ml) and H_2_O(4ml) was placed in a teflon reactor and heated at 80°C for 48h. After cooling to room temperature, colorless crystals suitable for X-ray diffraction were obtained with 42% yield based on Zn(NO_3_)_2_.6H_2_O.

### Refinement

All H atoms were placed in geometrically idealized positions (O—H 0.82, C—H 0.97(methylene), C—H 0.93(imidazolyl) C—H 0.93(naphthyl)Å) and treated as riding on their parent atoms, with *U*_iso_(H) = 1.5*U*_eq_(O), *U*_iso_(H) = 1.2*U*_eq_(C).

### Crystal data

**Table:**

[Zn(C_23_H_14_O_6_)(C_14_H_14_N_4_)]	*F*(000) = 1424
*M**_r_* = 690.00	*D*_x_ = 1.395 Mg m^−^^3^
Monoclinic, *P*2_1_/*c*	Mo *K*α radiation, λ = 0.71073 Å
Hall symbol: -P 2ybc	Cell parameters from 1774 reflections
*a* = 10.8382 (9) Å	θ = 2.3–26.1°
*b* = 17.3428 (16) Å	µ = 0.80 mm^−^^1^
*c* = 17.7939 (17) Å	*T* = 298 K
β = 100.781 (1)°	Block, colorless
*V* = 3285.6 (5) Å^3^	0.23 × 0.17 × 0.15 mm
*Z* = 4	

### Data collection

**Table:**

Bruker SMART-1000 CCD diffractometer	5800 independent reflections
Radiation source: fine-focus sealed tube	2460 reflections with *I* > 2σ(*I*)
graphite	*R*_int_ = 0.111
phi and ω scans	θ_max_ = 25.0°, θ_min_ = 2.2°
Absorption correction: multi-scan (*SADABS*; Sheldrick, 1996)	*h* = −7→12
*T*_min_ = 0.837, *T*_max_ = 0.889	*k* = −20→18
16511 measured reflections	*l* = −21→19

### Refinement

**Table:**

Refinement on *F*^2^	Primary atom site location: structure-invariant direct methods
Least-squares matrix: full	Secondary atom site location: difference Fourier map
*R*[*F*^2^ > 2σ(*F*^2^)] = 0.062	Hydrogen site location: inferred from neighbouring sites
*wR*(*F*^2^) = 0.127	H-atom parameters constrained
*S* = 1.00	*w* = 1/[σ^2^(*F*_o_^2^) + (0.0236*P*)^2^] where *P* = (*F*_o_^2^ + 2*F*_c_^2^)/3
5800 reflections	(Δ/σ)_max_ = 0.001
433 parameters	Δρ_max_ = 0.67 e Å^−^^3^
0 restraints	Δρ_min_ = −0.67 e Å^−^^3^

### Fractional atomic coordinates and isotropic or equivalent isotropic displacement parameters (Å^2^)

**Table:**

	*x*	*y*	*z*	*U*_iso_*/*U*_eq_	
Zn1	−0.39449 (6)	0.50406 (4)	0.24221 (4)	0.0467 (2)	
O1	−0.3107 (4)	0.5943 (2)	0.2949 (2)	0.0604 (12)	
O2	−0.1690 (4)	0.5185 (2)	0.3671 (2)	0.0699 (12)	
O3	−0.0023 (4)	0.57744 (19)	0.4719 (2)	0.0619 (12)	
H3	−0.0409	0.5448	0.4434	0.093*	
O4	0.5308 (4)	0.9456 (2)	0.6440 (2)	0.0611 (12)	
O5	0.3738 (4)	0.9219 (2)	0.7034 (2)	0.0763 (14)	
O6	0.2189 (4)	0.8163 (2)	0.6615 (2)	0.0700 (13)	
H6	0.2553	0.8513	0.6876	0.105*	
N1	−0.1407 (4)	0.3384 (3)	0.1945 (3)	0.0495 (13)	
N2	−0.2782 (4)	0.4302 (3)	0.2033 (3)	0.0495 (13)	
N3	0.3730 (5)	0.4304 (3)	0.3875 (3)	0.0554 (13)	
N4	0.5190 (5)	0.4570 (3)	0.3194 (3)	0.0532 (13)	
C1	−0.2182 (6)	0.5840 (4)	0.3479 (3)	0.0505 (16)	
C2	0.4305 (7)	0.9104 (3)	0.6495 (4)	0.0503 (16)	
C3	0.1095 (5)	0.7031 (3)	0.5576 (3)	0.0495 (15)	
H3A	0.1300	0.6487	0.5626	0.059*	
H3B	0.0865	0.7195	0.6052	0.059*	
C4	−0.0044 (6)	0.7121 (3)	0.4946 (3)	0.0386 (12)	
C5	−0.0545 (5)	0.6475 (3)	0.4542 (3)	0.0446 (15)	
C6	−0.1615 (5)	0.6530 (3)	0.3926 (3)	0.0424 (14)	
C7	−0.2144 (5)	0.7256 (3)	0.3752 (3)	0.0503 (16)	
H7	−0.2815	0.7305	0.3344	0.060*	
C8	−0.1694 (6)	0.7915 (3)	0.4174 (3)	0.0525 (16)	
C9	−0.0664 (5)	0.7846 (3)	0.4788 (3)	0.0445 (15)	
C10	−0.0301 (6)	0.8524 (3)	0.5231 (3)	0.0536 (17)	
H10	0.0375	0.8493	0.5638	0.064*	
C11	−0.0892 (7)	0.9204 (4)	0.5084 (4)	0.070 (2)	
H11	−0.0649	0.9625	0.5402	0.084*	
C12	−0.1880 (8)	0.9278 (4)	0.4450 (5)	0.080 (2)	
H12	−0.2263	0.9755	0.4338	0.096*	
C13	−0.2281 (6)	0.8654 (4)	0.3996 (4)	0.0688 (19)	
H13	−0.2929	0.8708	0.3576	0.083*	
C14	0.2276 (5)	0.7466 (3)	0.5483 (3)	0.0448 (15)	
C15	0.2754 (6)	0.8037 (3)	0.5997 (3)	0.0468 (15)	
C16	0.3828 (5)	0.8488 (3)	0.5920 (3)	0.0443 (15)	
C17	0.4395 (5)	0.8354 (3)	0.5311 (3)	0.0511 (16)	
H17	0.5063	0.8666	0.5241	0.061*	
C18	0.3996 (5)	0.7754 (3)	0.4782 (3)	0.0486 (15)	
C19	0.2920 (5)	0.7298 (3)	0.4877 (3)	0.0431 (14)	
C20	0.2585 (6)	0.6664 (3)	0.4362 (3)	0.0530 (16)	
H20	0.1903	0.6353	0.4407	0.064*	
C21	0.3278 (6)	0.6513 (3)	0.3798 (3)	0.0605 (18)	
H21	0.3052	0.6097	0.3472	0.073*	
C22	0.4289 (7)	0.6959 (4)	0.3705 (4)	0.074 (2)	
H22	0.4727	0.6849	0.3316	0.088*	
C23	0.4644 (6)	0.7565 (4)	0.4189 (4)	0.0646 (18)	
H23	0.5332	0.7862	0.4125	0.077*	
C24	−0.2301 (6)	0.3633 (3)	0.2317 (3)	0.0512 (16)	
H24	−0.2551	0.3372	0.2720	0.061*	
C25	−0.2178 (7)	0.4463 (4)	0.1459 (4)	0.082 (2)	
H25	−0.2320	0.4898	0.1150	0.098*	
C26	−0.1329 (7)	0.3902 (4)	0.1392 (4)	0.081 (2)	
H26	−0.0802	0.3880	0.1036	0.098*	
C27	0.3969 (6)	0.4536 (3)	0.3211 (3)	0.0494 (15)	
H27	0.3345	0.4664	0.2796	0.059*	
C28	0.5744 (6)	0.4335 (4)	0.3922 (4)	0.071 (2)	
H28	0.6606	0.4293	0.4096	0.085*	
C29	0.4875 (7)	0.4179 (4)	0.4341 (4)	0.079 (2)	
H29	0.5015	0.4017	0.4848	0.095*	
C30	−0.0691 (6)	0.2657 (3)	0.2106 (3)	0.0576 (17)	
H30A	−0.0437	0.2486	0.1639	0.069*	
H30B	−0.1246	0.2267	0.2249	0.069*	
C31	0.2486 (6)	0.4144 (3)	0.4057 (3)	0.0644 (18)	
H31A	0.1919	0.4568	0.3884	0.077*	
H31B	0.2554	0.4094	0.4607	0.077*	
C32	0.0448 (6)	0.2705 (3)	0.2720 (3)	0.0489 (16)	
C33	0.0892 (6)	0.3386 (3)	0.3090 (3)	0.0545 (16)	
H33	0.0464	0.3845	0.2953	0.065*	
C34	0.1966 (6)	0.3394 (4)	0.3661 (4)	0.0567 (17)	
C35	0.2594 (6)	0.2702 (4)	0.3865 (4)	0.0680 (19)	
H35	0.3304	0.2691	0.4250	0.082*	
C36	0.2149 (7)	0.2026 (4)	0.3485 (4)	0.070 (2)	
H36	0.2580	0.1566	0.3610	0.084*	
C37	0.1091 (6)	0.2030 (4)	0.2934 (4)	0.0598 (18)	
H37	0.0797	0.1569	0.2699	0.072*	

### Atomic displacement parameters (Å^2^)

**Table:**

	*U*^11^	*U*^22^	*U*^33^	*U*^12^	*U*^13^	*U*^23^
Zn1	0.0379 (4)	0.0440 (4)	0.0570 (4)	0.0013 (4)	0.0059 (3)	0.0023 (4)
O1	0.053 (3)	0.063 (3)	0.060 (3)	−0.009 (2)	−0.002 (2)	0.000 (2)
O2	0.064 (3)	0.047 (3)	0.089 (3)	−0.008 (2)	−0.012 (2)	−0.006 (2)
O3	0.056 (3)	0.029 (2)	0.091 (3)	−0.004 (2)	−0.012 (2)	0.001 (2)
O4	0.057 (3)	0.054 (3)	0.068 (3)	−0.011 (2)	0.001 (2)	−0.001 (2)
O5	0.086 (4)	0.079 (3)	0.064 (3)	−0.025 (3)	0.014 (3)	−0.026 (3)
O6	0.075 (3)	0.084 (3)	0.055 (3)	−0.031 (3)	0.022 (3)	−0.019 (2)
N1	0.041 (3)	0.049 (3)	0.060 (3)	0.011 (3)	0.013 (3)	0.004 (3)
N2	0.045 (3)	0.051 (3)	0.053 (3)	0.009 (3)	0.010 (3)	0.008 (3)
N3	0.044 (4)	0.062 (3)	0.062 (4)	−0.005 (3)	0.013 (3)	0.003 (3)
N4	0.048 (4)	0.059 (3)	0.053 (3)	0.010 (3)	0.008 (3)	0.004 (3)
C1	0.048 (5)	0.048 (4)	0.057 (4)	−0.011 (4)	0.011 (3)	−0.002 (3)
C2	0.050 (5)	0.049 (4)	0.049 (4)	−0.002 (4)	0.003 (4)	0.002 (3)
C3	0.049 (4)	0.055 (4)	0.043 (4)	−0.004 (3)	0.003 (3)	−0.001 (3)
C4	0.034 (3)	0.042 (3)	0.041 (3)	−0.007 (3)	0.011 (3)	−0.005 (3)
C5	0.036 (4)	0.049 (4)	0.049 (4)	−0.007 (3)	0.009 (3)	0.007 (3)
C6	0.036 (4)	0.045 (3)	0.049 (4)	−0.007 (3)	0.016 (3)	0.005 (3)
C7	0.032 (4)	0.064 (4)	0.056 (4)	−0.007 (3)	0.009 (3)	0.006 (3)
C8	0.045 (4)	0.046 (4)	0.072 (5)	−0.006 (3)	0.024 (4)	−0.001 (3)
C9	0.032 (4)	0.044 (4)	0.060 (4)	−0.010 (3)	0.018 (3)	0.000 (3)
C10	0.048 (4)	0.053 (4)	0.063 (4)	−0.008 (4)	0.018 (3)	−0.009 (3)
C11	0.077 (6)	0.042 (4)	0.096 (6)	−0.012 (4)	0.031 (5)	−0.012 (4)
C12	0.077 (6)	0.039 (4)	0.129 (7)	0.005 (4)	0.036 (5)	−0.002 (4)
C13	0.052 (5)	0.060 (4)	0.096 (5)	0.011 (4)	0.018 (4)	0.019 (4)
C14	0.041 (4)	0.049 (4)	0.046 (4)	−0.005 (3)	0.013 (3)	−0.001 (3)
C15	0.048 (4)	0.053 (4)	0.040 (4)	−0.012 (3)	0.012 (3)	−0.004 (3)
C16	0.043 (4)	0.043 (3)	0.044 (4)	−0.001 (3)	0.000 (3)	−0.002 (3)
C17	0.037 (4)	0.052 (4)	0.061 (4)	−0.008 (3)	0.003 (3)	0.004 (3)
C18	0.039 (4)	0.055 (4)	0.050 (4)	−0.001 (3)	0.006 (3)	−0.010 (3)
C19	0.033 (4)	0.042 (3)	0.052 (4)	0.001 (3)	0.001 (3)	−0.002 (3)
C20	0.051 (4)	0.051 (4)	0.054 (4)	0.003 (3)	0.000 (3)	−0.009 (3)
C21	0.054 (5)	0.065 (4)	0.059 (4)	0.000 (4)	0.003 (4)	−0.018 (4)
C22	0.059 (5)	0.100 (6)	0.065 (5)	0.000 (5)	0.021 (4)	−0.014 (4)
C23	0.052 (5)	0.074 (5)	0.070 (5)	−0.006 (4)	0.015 (4)	−0.005 (4)
C24	0.045 (4)	0.054 (4)	0.057 (4)	0.004 (3)	0.016 (3)	0.007 (3)
C25	0.104 (7)	0.070 (5)	0.083 (5)	0.026 (5)	0.047 (5)	0.028 (4)
C26	0.091 (6)	0.082 (5)	0.086 (5)	0.027 (5)	0.056 (5)	0.023 (4)
C27	0.043 (4)	0.056 (4)	0.049 (4)	−0.002 (3)	0.006 (3)	0.010 (3)
C28	0.033 (4)	0.091 (5)	0.088 (5)	−0.007 (4)	0.010 (4)	0.027 (4)
C29	0.060 (5)	0.105 (6)	0.066 (5)	−0.004 (5)	−0.005 (4)	0.025 (4)
C30	0.054 (5)	0.049 (4)	0.071 (4)	0.010 (3)	0.014 (4)	−0.003 (3)
C31	0.048 (5)	0.070 (5)	0.077 (5)	0.003 (4)	0.015 (4)	0.006 (4)
C32	0.042 (4)	0.052 (4)	0.054 (4)	0.006 (3)	0.014 (3)	0.005 (3)
C33	0.040 (4)	0.048 (4)	0.079 (5)	0.003 (3)	0.019 (4)	0.002 (4)
C34	0.049 (5)	0.055 (4)	0.071 (5)	−0.001 (4)	0.025 (4)	0.001 (4)
C35	0.055 (5)	0.074 (5)	0.073 (5)	0.005 (4)	0.009 (4)	0.018 (4)
C36	0.061 (5)	0.051 (4)	0.101 (6)	0.016 (4)	0.021 (5)	0.016 (4)
C37	0.058 (5)	0.051 (4)	0.072 (5)	0.005 (4)	0.018 (4)	0.006 (4)

### Geometric parameters (Å, °)

**Table:**

Zn1—O1	1.957 (4)	C12—C13	1.371 (8)
Zn1—N4^i^	1.979 (5)	C12—H12	0.9300
Zn1—O4^ii^	1.985 (4)	C13—H13	0.9300
Zn1—N2	2.009 (5)	C14—C15	1.382 (7)
O1—C1	1.255 (7)	C14—C19	1.418 (7)
O2—C1	1.274 (6)	C15—C16	1.430 (7)
O3—C5	1.352 (6)	C16—C17	1.362 (7)
O3—H3	0.8200	C17—C18	1.415 (7)
O4—C2	1.267 (6)	C17—H17	0.9300
O4—Zn1^iii^	1.985 (4)	C18—C23	1.412 (7)
O5—C2	1.248 (6)	C18—C19	1.445 (7)
O6—C15	1.372 (5)	C19—C20	1.435 (7)
O6—H6	0.8200	C20—C21	1.386 (7)
N1—C24	1.342 (6)	C20—H20	0.9300
N1—C26	1.346 (6)	C21—C22	1.376 (8)
N1—C30	1.480 (6)	C21—H21	0.9300
N2—C24	1.332 (6)	C22—C23	1.368 (8)
N2—C25	1.342 (6)	C22—H22	0.9300
N3—C27	1.318 (6)	C23—H23	0.9300
N3—C29	1.374 (7)	C24—H24	0.9300
N3—C31	1.471 (7)	C25—C26	1.359 (8)
N4—C27	1.331 (6)	C25—H25	0.9300
N4—C28	1.383 (7)	C26—H26	0.9300
N4—Zn1^iv^	1.979 (5)	C27—H27	0.9300
C1—C6	1.503 (7)	C28—C29	1.335 (8)
C2—C16	1.502 (7)	C28—H28	0.9300
C3—C4	1.512 (7)	C29—H29	0.9300
C3—C14	1.521 (7)	C30—C32	1.490 (7)
C3—H3A	0.9700	C30—H30A	0.9700
C3—H3B	0.9700	C30—H30B	0.9700
C4—C5	1.387 (7)	C31—C34	1.535 (8)
C4—C9	1.429 (7)	C31—H31A	0.9700
C5—C6	1.442 (7)	C31—H31B	0.9700
C6—C7	1.393 (7)	C32—C37	1.379 (7)
C7—C8	1.405 (7)	C32—C33	1.394 (7)
C7—H7	0.9300	C33—C34	1.395 (8)
C8—C9	1.414 (7)	C33—H33	0.9300
C8—C13	1.440 (7)	C34—C35	1.395 (8)
C9—C10	1.428 (7)	C35—C36	1.393 (8)
C10—C11	1.344 (8)	C35—H35	0.9300
C10—H10	0.9300	C36—C37	1.362 (8)
C11—C12	1.409 (9)	C36—H36	0.9300
C11—H11	0.9300	C37—H37	0.9300
			
O1—Zn1—N4^i^	103.57 (17)	C17—C16—C2	120.9 (6)
O1—Zn1—O4^ii^	98.40 (16)	C15—C16—C2	120.4 (5)
N4^i^—Zn1—O4^ii^	128.6 (2)	C16—C17—C18	121.8 (5)
O1—Zn1—N2	114.19 (19)	C16—C17—H17	119.1
N4^i^—Zn1—N2	112.68 (19)	C18—C17—H17	119.1
O4^ii^—Zn1—N2	99.00 (18)	C23—C18—C17	122.3 (6)
C1—O1—Zn1	118.7 (4)	C23—C18—C19	119.1 (5)
C5—O3—H3	109.5	C17—C18—C19	118.5 (5)
C2—O4—Zn1^iii^	111.1 (4)	C14—C19—C20	122.7 (5)
C15—O6—H6	109.5	C14—C19—C18	119.9 (5)
C24—N1—C26	107.2 (5)	C20—C19—C18	117.3 (5)
C24—N1—C30	125.3 (5)	C21—C20—C19	120.0 (6)
C26—N1—C30	127.4 (5)	C21—C20—H20	120.0
C24—N2—C25	104.9 (5)	C19—C20—H20	120.0
C24—N2—Zn1	130.7 (4)	C22—C21—C20	122.2 (6)
C25—N2—Zn1	123.8 (4)	C22—C21—H21	118.9
C27—N3—C29	106.4 (5)	C20—C21—H21	118.9
C27—N3—C31	126.6 (6)	C23—C22—C21	119.5 (6)
C29—N3—C31	126.7 (6)	C23—C22—H22	120.2
C27—N4—C28	103.0 (5)	C21—C22—H22	120.2
C27—N4—Zn1^iv^	129.5 (4)	C22—C23—C18	121.9 (6)
C28—N4—Zn1^iv^	126.3 (4)	C22—C23—H23	119.1
O1—C1—O2	124.3 (6)	C18—C23—H23	119.1
O1—C1—C6	118.3 (6)	N2—C24—N1	111.1 (5)
O2—C1—C6	117.3 (6)	N2—C24—H24	124.4
O5—C2—O4	122.3 (6)	N1—C24—H24	124.4
O5—C2—C16	118.8 (6)	N2—C25—C26	110.6 (6)
O4—C2—C16	118.8 (6)	N2—C25—H25	124.7
C4—C3—C14	117.1 (4)	C26—C25—H25	124.7
C4—C3—H3A	108.0	N1—C26—C25	106.1 (5)
C14—C3—H3A	108.0	N1—C26—H26	127.0
C4—C3—H3B	108.0	C25—C26—H26	127.0
C14—C3—H3B	108.0	N3—C27—N4	113.3 (5)
H3A—C3—H3B	107.3	N3—C27—H27	123.3
C5—C4—C9	119.0 (6)	N4—C27—H27	123.3
C5—C4—C3	119.2 (5)	C29—C28—N4	110.8 (6)
C9—C4—C3	121.7 (5)	C29—C28—H28	124.6
O3—C5—C4	119.9 (5)	N4—C28—H28	124.6
O3—C5—C6	118.6 (5)	C28—C29—N3	106.4 (6)
C4—C5—C6	121.4 (5)	C28—C29—H29	126.8
C7—C6—C5	117.9 (5)	N3—C29—H29	126.8
C7—C6—C1	119.4 (5)	N1—C30—C32	115.3 (5)
C5—C6—C1	122.7 (5)	N1—C30—H30A	108.4
C6—C7—C8	121.9 (6)	C32—C30—H30A	108.4
C6—C7—H7	119.0	N1—C30—H30B	108.4
C8—C7—H7	119.0	C32—C30—H30B	108.4
C7—C8—C9	119.3 (5)	H30A—C30—H30B	107.5
C7—C8—C13	120.8 (6)	N3—C31—C34	109.3 (5)
C9—C8—C13	119.9 (6)	N3—C31—H31A	109.8
C8—C9—C10	116.9 (5)	C34—C31—H31A	109.8
C8—C9—C4	120.1 (5)	N3—C31—H31B	109.8
C10—C9—C4	123.0 (6)	C34—C31—H31B	109.8
C11—C10—C9	122.9 (6)	H31A—C31—H31B	108.3
C11—C10—H10	118.6	C37—C32—C33	118.5 (6)
C9—C10—H10	118.6	C37—C32—C30	117.5 (6)
C10—C11—C12	119.9 (6)	C33—C32—C30	124.0 (6)
C10—C11—H11	120.0	C32—C33—C34	121.3 (6)
C12—C11—H11	120.0	C32—C33—H33	119.3
C13—C12—C11	120.6 (7)	C34—C33—H33	119.3
C13—C12—H12	119.7	C35—C34—C33	118.7 (6)
C11—C12—H12	119.7	C35—C34—C31	119.4 (6)
C12—C13—C8	119.7 (7)	C33—C34—C31	121.8 (6)
C12—C13—H13	120.2	C36—C35—C34	119.4 (6)
C8—C13—H13	120.2	C36—C35—H35	120.3
C15—C14—C19	118.3 (5)	C34—C35—H35	120.3
C15—C14—C3	120.1 (5)	C37—C36—C35	120.9 (6)
C19—C14—C3	121.6 (5)	C37—C36—H36	119.5
O6—C15—C14	118.5 (5)	C35—C36—H36	119.5
O6—C15—C16	118.9 (5)	C36—C37—C32	121.1 (6)
C14—C15—C16	122.6 (5)	C36—C37—H37	119.5
C17—C16—C15	118.7 (5)	C32—C37—H37	119.5
			
N4^i^—Zn1—O1—C1	71.0 (5)	O5—C2—C16—C15	−0.8 (8)
O4^ii^—Zn1—O1—C1	−155.8 (4)	O4—C2—C16—C15	175.1 (5)
N2—Zn1—O1—C1	−51.9 (5)	C15—C16—C17—C18	−3.8 (8)
O1—Zn1—N2—C24	101.2 (5)	C2—C16—C17—C18	177.1 (5)
N4^i^—Zn1—N2—C24	−16.6 (6)	C16—C17—C18—C23	−174.2 (5)
O4^ii^—Zn1—N2—C24	−155.3 (5)	C16—C17—C18—C19	3.0 (8)
O1—Zn1—N2—C25	−68.4 (5)	C15—C14—C19—C20	173.1 (5)
N4^i^—Zn1—N2—C25	173.8 (5)	C3—C14—C19—C20	−6.5 (8)
O4^ii^—Zn1—N2—C25	35.1 (5)	C15—C14—C19—C18	−4.0 (8)
Zn1—O1—C1—O2	4.7 (8)	C3—C14—C19—C18	176.4 (5)
Zn1—O1—C1—C6	−174.7 (3)	C23—C18—C19—C14	178.3 (5)
Zn1^iii^—O4—C2—O5	15.3 (7)	C17—C18—C19—C14	1.0 (8)
Zn1^iii^—O4—C2—C16	−160.4 (4)	C23—C18—C19—C20	1.1 (8)
C14—C3—C4—C5	119.1 (6)	C17—C18—C19—C20	−176.2 (5)
C14—C3—C4—C9	−64.8 (7)	C14—C19—C20—C21	−177.7 (5)
C9—C4—C5—O3	−175.4 (5)	C18—C19—C20—C21	−0.6 (8)
C3—C4—C5—O3	0.8 (8)	C19—C20—C21—C22	−0.5 (9)
C9—C4—C5—C6	5.0 (8)	C20—C21—C22—C23	1.1 (10)
C3—C4—C5—C6	−178.8 (5)	C21—C22—C23—C18	−0.5 (10)
O3—C5—C6—C7	179.8 (5)	C17—C18—C23—C22	176.6 (6)
C4—C5—C6—C7	−0.6 (8)	C19—C18—C23—C22	−0.6 (9)
O3—C5—C6—C1	0.6 (8)	C25—N2—C24—N1	1.1 (7)
C4—C5—C6—C1	−179.8 (5)	Zn1—N2—C24—N1	−170.0 (4)
O1—C1—C6—C7	1.3 (8)	C26—N1—C24—N2	−1.4 (7)
O2—C1—C6—C7	−178.1 (5)	C30—N1—C24—N2	−179.2 (5)
O1—C1—C6—C5	−179.5 (5)	C24—N2—C25—C26	−0.3 (8)
O2—C1—C6—C5	1.1 (8)	Zn1—N2—C25—C26	171.5 (5)
C5—C6—C7—C8	−2.4 (8)	C24—N1—C26—C25	1.1 (8)
C1—C6—C7—C8	176.8 (5)	C30—N1—C26—C25	178.9 (6)
C6—C7—C8—C9	0.8 (8)	N2—C25—C26—N1	−0.5 (9)
C6—C7—C8—C13	−178.1 (5)	C29—N3—C27—N4	0.6 (7)
C7—C8—C9—C10	−175.8 (5)	C31—N3—C27—N4	−174.6 (5)
C13—C8—C9—C10	3.1 (8)	C28—N4—C27—N3	−0.1 (7)
C7—C8—C9—C4	3.8 (8)	Zn1^iv^—N4—C27—N3	−168.4 (4)
C13—C8—C9—C4	−177.3 (5)	C27—N4—C28—C29	−0.5 (7)
C5—C4—C9—C8	−6.6 (8)	Zn1^iv^—N4—C28—C29	168.4 (4)
C3—C4—C9—C8	177.3 (5)	N4—C28—C29—N3	0.8 (8)
C5—C4—C9—C10	172.9 (5)	C27—N3—C29—C28	−0.8 (7)
C3—C4—C9—C10	−3.2 (8)	C31—N3—C29—C28	174.3 (6)
C8—C9—C10—C11	0.1 (8)	C24—N1—C30—C32	−86.1 (7)
C4—C9—C10—C11	−179.5 (5)	C26—N1—C30—C32	96.6 (7)
C9—C10—C11—C12	−3.0 (9)	C27—N3—C31—C34	72.1 (7)
C10—C11—C12—C13	2.7 (10)	C29—N3—C31—C34	−102.1 (7)
C11—C12—C13—C8	0.5 (10)	N1—C30—C32—C37	174.9 (5)
C7—C8—C13—C12	175.5 (6)	N1—C30—C32—C33	−4.6 (8)
C9—C8—C13—C12	−3.4 (9)	C37—C32—C33—C34	0.4 (8)
C4—C3—C14—C15	116.3 (6)	C30—C32—C33—C34	180.0 (5)
C4—C3—C14—C19	−64.1 (7)	C32—C33—C34—C35	−0.5 (9)
C19—C14—C15—O6	−175.4 (5)	C32—C33—C34—C31	178.2 (5)
C3—C14—C15—O6	4.2 (8)	N3—C31—C34—C35	63.5 (7)
C19—C14—C15—C16	3.2 (9)	N3—C31—C34—C33	−115.1 (6)
C3—C14—C15—C16	−177.2 (5)	C33—C34—C35—C36	1.1 (9)
O6—C15—C16—C17	179.3 (5)	C31—C34—C35—C36	−177.6 (5)
C14—C15—C16—C17	0.6 (9)	C34—C35—C36—C37	−1.8 (10)
O6—C15—C16—C2	−1.6 (8)	C35—C36—C37—C32	1.8 (10)
C14—C15—C16—C2	179.7 (5)	C33—C32—C37—C36	−1.1 (9)
O5—C2—C16—C17	178.3 (5)	C30—C32—C37—C36	179.3 (5)
O4—C2—C16—C17	−5.8 (8)		

Symmetry codes: (i) *x*−1, *y*, *z*; (ii) *x*−1, −*y*+3/2, *z*−1/2; (iii) *x*+1, −*y*+3/2, *z*+1/2; (iv) *x*+1, *y*, *z*.

### Hydrogen-bond geometry (Å, °)

**Table:**

Cg1, Cg2 and Cg3 are the centroids of the C8–C13, C18–C23 and C4–C9 rings, respectively.

**Table:**

*D*—H···*A*	*D*—H	H···*A*	*D*···*A*	*D*—H···*A*
O3—H3···O2	0.82	1.81	2.555 (5)	150
O6—H6···O5	0.82	1.76	2.502 (5)	150
C30—H30B···O6^v^	0.97	2.54	3.350 (7)	141
C26—H26···Cg1^vi^	0.93	2.79	3.676 (8)	161
C29—H29···Cg2^vii^	0.93	2.76	3.502 (7)	137
C30—H30A···Cg3^vi^	0.97	2.71	3.628 (6)	158

Symmetry codes: (v) −*x*, −*y*+1, −*z*+1; (vi) −*x*, *y*−1/2, −*z*+1/2; (vii) −*x*+1, −*y*+1, −*z*+1.
